# Healthcare Utilization and Costs of Systemic Lupus Erythematosus in Medicaid

**DOI:** 10.1155/2013/808391

**Published:** 2012-12-05

**Authors:** Hong J. Kan, Xue Song, Barbara H. Johnson, Benno Bechtel, Donna O'Sullivan, Charles T. Molta

**Affiliations:** ^1^U.S. Health Outcomes, GlaxoSmithKline, Research Triangle Park, NC 27709, USA; ^2^Pharma, Truven Health Analytics Inc., Cambridge, MA 02140, USA; ^3^Pharma, Truven Health Analytics Inc., Washington, DC 20008, USA; ^4^European Market Access at Critical Disease Business Unit, GlaxoSmithKline, London TW8 9GS, UK; ^5^Global Medical Affairs, GlaxoSmithKline, Philadelphia, PA 19102, USA

## Abstract

*Objective*. Healthcare utilization and costs associated with systemic lupus erythematosus (SLE) in a US Medicaid population were examined. *Methods*. Patients ≥ 18 years old with SLE diagnosis (ICD-9-CM 710.0x) were extracted from a large Medicaid database 2002–2009. Index date was date of the first SLE diagnosis. Patients with and without SLE were matched. All patients had a variable length of followup with a minimum of 12 months. Annualized healthcare utilization and costs associated with SLE and costs of SLE flares were assessed during the followup period. Multivariate regressions were conducted to estimate incremental healthcare utilization and costs associated with SLE. *Results*. A total of 14,777 SLE patients met the study criteria, and 14,262 were matched to non-SLE patients. SLE patients had significantly higher healthcare utilization per year than their matched controls. The estimated incremental annual cost associated with SLE was $10,984, with the highest increase in inpatient costs (*P* < 0.001). Cost per flare was $11,716 for severe flares, $562 for moderate flares, and $129 for mild flares. Annual total costs for patients with severe flares were $49,754. *Conclusions*. SLE patients had significantly higher healthcare resource utilization and costs than non-SLE patients. Patients with severe flares had the highest costs.

## 1. Introduction

Systemic lupus erythematosus (SLE) is a chronic autoimmune disease with a variety of clinical manifestations and autoantibodies [1]. It is estimated that 161,000 to 322,000 people in the US have been diagnosed with SLE [2, 3]. The Lupus Foundation of America (LFA) estimates that more than 90% of affected patients are women, most often between the ages of 15 and 45 [4]. In the US, minority populations, especially African Americans and Hispanics and people of lower socioeconomic status, have a higher overall prevalence of SLE [5]. 

The course of SLE is unpredictable, with periods of flares alternating with periods of less severepersistent disease activity. There is currently no cure for SLE. Corticosteroids, antimalarials, and immunosuppressants have been the main immunomodulatory medications used for pharmacological therapy. Biological drugs that target B-cells or specific pathways (i.e., T-B lymphocyte interaction, cytokines, and complement) have been evaluated as new SLE treatment [6].

The management of SLE is costly. A study of a commercial population estimated a mean annual medical cost of $12,238 higher (2005 dollars) in SLE patients than matched non-SLE patients [7]. Another Medicaid study estimated a medical cost of $6,831 higher than matched non-SLE patients during the first year of SLE diagnosis and $8,189 higher during the fifth year (2006 dollars) [8]. These two studies used 2000–2004 data and 1999–2005 data, respectively. Neither study examined SLE flares nor the costs associated with flares. This study used more recent administrative claims data to estimate healthcare utilization and costs associated with SLE and examined cost of flares in a prevalent SLE population in Medicaid.

## 2. Materials and Methods

### 2.1. Data Source

This study used the *Truven Health MarketScan Multi-State Medicaid Database* for patients enrolled in 2002–2009. The database contains pooled healthcare experience of nearly 30 million Medicaid enrollees from 10 geographically dispersed states. Enrollees in the database are covered under both fee-for-service and managed care plans. This claims database is constructed from paid medical and prescription drug claims that have been de-identified and standardized for research purposes. It provides detailed utilization, expenditure, and outcomes data for healthcare services performed in both inpatient and outpatient settings. The medical claims are linked to outpatient prescription drug claims and person-level enrollment data through the use of unique enrollee identifiers. Data are fully compliant with the Health Insurance Portability and Accountability Act of 1996. 

### 2.2. Study Population

SLE patients were required to have at least one inpatient SLE diagnosis code (ICD-9-CM 710.0x) in any position on the claim or at least two non-diagnostic (not laboratory or radiology) outpatient claims at least 30 days but less than 2 years apart with an SLE diagnosis code in any position. The date of the first SLE diagnosis in 2003–2008 was set as the index date. All patients were required to be at least 18 years old on index date, having continuous eligibility in the database with both medical and pharmaceutical benefits at least six months before (pre-period) and 12 months following (followup period) the index date. The length of followup period was variable but being at least 12 months. Patients were followed from index date to the earliest of inpatient death, end of continuous enrollment, or end of the study period (12/31/2009).

### 2.3. Variable Definitions at Patient Level

Demographic characteristics measured on index date included age, gender, plan type, Medicare dual eligibility, reasons for Medicaid eligibility, race, index year, and length of followup period. Clinical characteristics were measured in the pre-period and included the Deyo adaptation of the Charlson comorbidity index (CCI) as an overall measure of burden of illness [9]. Evidence of selected comorbid conditions (i.e., rheumatoid arthritis and other inflammatory polyarthropathies, autoimmune thyroid disorders, anemia, pericarditis, Raynaud's syndrome, thrombocytopenia, myositis, hypertension, renal disease, depression, cardiac disease, cerebrovascular disease, liver disease, pulmonary disease, and nephritis) and the use of selected concomitant medications that might trigger the development of SLE (i.e., hydralazine, quinidine, procainamide, phenytoin, isoniazid, d-penicillamine) were recorded.

Healthcare utilization was measured during the followup period. Specific utilization measures included inpatient hospitalizations, emergency room (ER) visits, physician office visits, hospital-based outpatient visits, other outpatient services (including laboratory, radiology, and therapies), and use of SLE medications (i.e., nonsteroidal anti-inflammatory drugs, corticosteroids, antimalarials, immunosuppressives, androgens, and rituximab).

Total healthcare costs, regardless of whether they were associated with SLE, were measured during the entire followup period and were broken down by inpatient, outpatient (including ER visits, outpatient physician office visits, outpatient hospital, and outpatient other), and total outpatient pharmacy. Costs were the total reimbursed amount, including patient co-pay and deductibles. All costs were inflated to 2009 dollars using the medical component of Consumer Price Index. Because of the variable length of followup, utilization and cost measures were standardized as annual utilization and costs.

### 2.4. Flare Episodes and Costs Per Flare

In addition to all medical costs at the patient level, this study also examined costs of treating flares during each flare episode. An algorithm to define SLE flare episodes was developed using the framework from the Lupus Foundation of America Second International Lupus Flare Conference [10] and criteria from the British Isles Lupus Assessment Group (BILAG) index [11].

Flare episodes were identified by flare severity (mild, moderate, and severe). Mild flare episodes were defined as beginning with the initiation of hydroxychloroquine or another antimalarial, an oral corticosteroid with prednisone-equivalent dose of ≤7.5 mg/day, or nonimmunosuppressive therapy (NSAIDS, androgens). Moderate flare episodes begun with the initiation of an oral corticosteroid with prednisone-equivalent dose >7.5 mg/day but ≤40 mg/day or immunosuppressive therapy, with the exception of cyclophosphamide, or a claim for an ER visit with a primary diagnosis of SLE with no inpatient admission within 1 day, or a claim for an ER or office visit with a primary or secondary diagnosis for a specified SLE-related condition. Severe flare episodes began with the initiation of an oral corticosteroid with prednisone-equivalent dose >40 mg/day or cylophosphamide, or admission for an inpatient hospital stay with a primary diagnosis of SLE or a specified SLE-related condition. Duration of each flare episode was set to 30 days by default. However, if a flare of higher severity occurred during those 30 days, the length of the flare episode was limited to the time between the start of the original flare episode and the start of the flare episode of higher severity [12, 13]. 

Costs of flare treatment were measured within each flare episode. Costs of mild flare episodes included only costs attributable to mild flare during that flare episode. Costs of moderate flare episodes included costs attributable to both mild and moderate flares beginning from the start to the end of the moderate flare episode. Costs of severe flare episodes included costs attributable to all three levels of flares beginning from the start of the severe flare episode and ending after 30 days. However, if the trigger of the severe flare episode was an inpatient hospitalization, the cost of the entire hospitalization was included in the flare episode costs even if the discharge date fell outside the flare episode.

### 2.5. Control Selection and Propensity Score Matching

A random sample of 10% from all adult patients in the database without an SLE diagnosis anytime between 2002 and 2009 was selected as the potential control cohort. The index date of the control patients was randomly assigned based on the distribution of index dates of SLE patients. Thus SLE patients and their controls had similar distribution in the number of days between index date and January 1, 2003. Potential controls were then screened for 6 months of continuous eligibility with medical and pharmaceutical benefits prior and 12 months subsequent to their respective index dates.

Propensity score analysis was performed to adjust for differences in patient profiles which can confound healthcare utilization and cost [14]. Matching factors included age, gender, race, urban residence, health plan type, Medicaid eligibility category, index year, Medicare dual eligibility, CCI, prevalence of comorbid conditions, and any use of concomitant medication that might trigger the development of SLE. SLE patients were matched to non-SLE patients using the nearest neighbor with 1 : 1 matching technique with caliper. Propensity score matching was conducted separately for patients in each contributing Medicaid state. 

Standardized differences were calculated to examine the quality of the match. It is considered a good match when the absolute value of standardized difference is less than 10 for the majority of matching factors [15].

### 2.6. Statistical Analyses

SAS 9.2 [16] was used to build the analytic file and conduct descriptive analysis. Stata 11 [17] was used to conduct propensity score matching and multivariate adjustment. Demographic and clinical characteristics and healthcare utilization and expenditures were reported for SLE patients and their matched non-SLE patients separately. Statistical tests of significance for differences in these distributions were conducted between SLE and non-SLE patients. *Z*-tests were used to evaluate equality of proportions for categorical variables, and *t*-tests were used for continuous variables. Number of flares and flare-related costs were also reported for SLE patients. 

Multivariate adjustment was conducted on the propensity score-matched sample to increase estimating efficiency and to control any remaining imbalances in observed covariates that affected the outcome estimates. Multivariate analysis also allowed us to estimate the marginal impact of SLE on healthcare utilization and costs. Logistic models were used to estimate whether a patient had at least one inpatient admission or at least one ER visit. Ordinary least squares (OLS) models were used to estimate number of inpatient admissions, physician office visits, ER visits, hospital outpatient visits, and other outpatient services. Generalized linear models (GLMs) with log link and gamma distribution were used to estimate total cost and cost components (inpatient, outpatient, and outpatient pharmacy). All matching factors plus an SLE indicator were included in the models as independent variables. 

## 3. Results

### 3.1. Demographic and Clinical Characteristics

**Figure 1:**
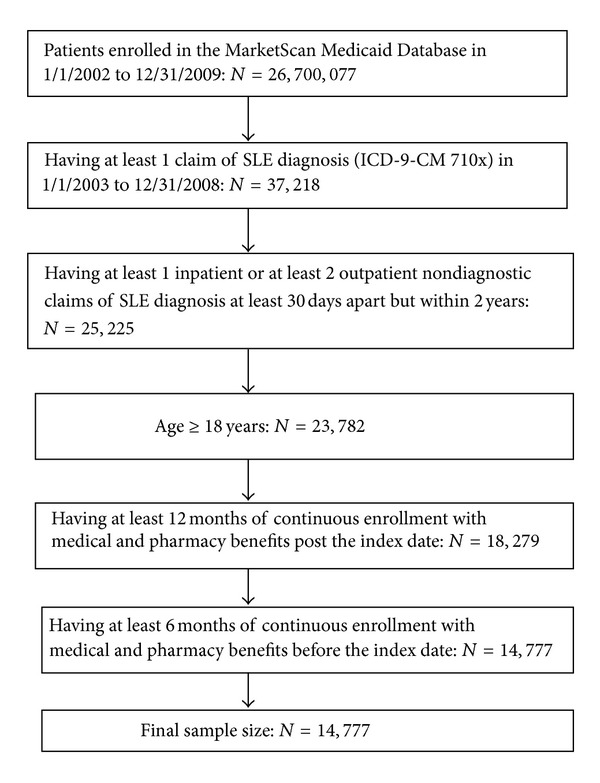
Sample Selection.

A total of 14,777 patients with evidence of SLE met the study inclusion criteria (Figure 1). Of those, 92.8% were women, and the mean age was 45.4 years (SD = 14.3, Table 1). The mean length of followup was 38.8 months (SD = 20.4). Nearly 40% of the sample were white, 36.6% African Americans, 11.4% Hispanics, and the remaining were other or unknown races. The most common comorbidities in the SLE population included hypertension (30.2%), cardiac disease (24.3%), pulmonary disease (17.5%), depression (15.7%), anemia (14.2%), rheumatoid arthritis (10.6%), and myositis (9.8%). A total of 14,262 patients with SLE were matched to patients without SLE. 

Before matching, SLE patients were significantly sicker than non-SLE patients, with a higher CCI (1.16 versus 0.41) and higher rates of comorbid conditions such as rheumatoid arthritis, anemia, myositis, hypertension, renal disease, depression, cardiac disease, and pulmonary disease. After matching, only age had an absolute value of standardized difference greater than 10 when compared to controls. All other variables were well matched.

### 3.2. Healthcare Utilization and Costs

Without exception, SLE patients used significantly more healthcare services than their matched controls during the followup period. Twenty percent more SLE patients had at least one inpatient admission, and 11% more SLE patients had at least one ER visit relative to their matched controls. They also had 3.4 more physician office visits, 1.1 more outpatient hospital visits, and 12.9 more other outpatient services per year than the non-SLE cohort (*P* < 0.001 in all cases, Table 2).

**Figure 2:**
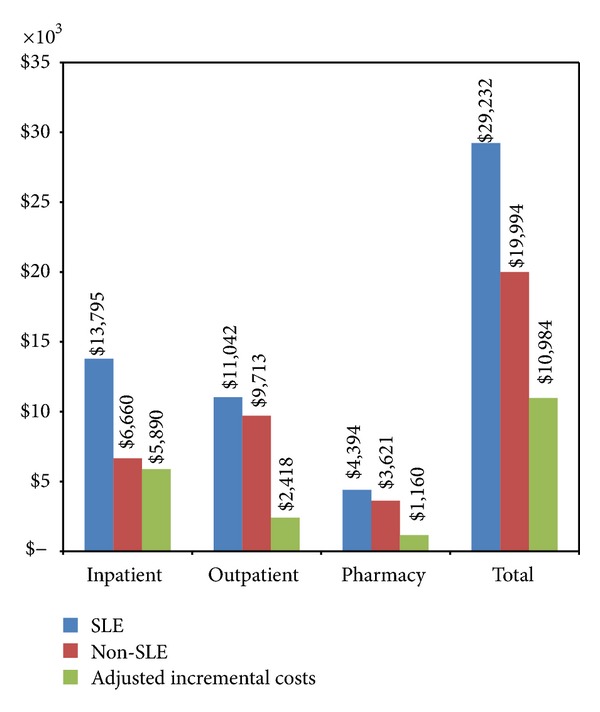
Annualized healthcare costs and multivariate regression adjusted annual incremental costs associated with SLE. *P* < 0.001 in all cases.

Total cost and cost components of SLE patients and their matched controls were reported in Figure 2. Inpatient and outpatient costs were the dominant cost drivers for both cohorts, consisting of 47% and 38% of total cost for SLE patients and 33% and 49% for non-SLE patients, respectively. Consistent with the utilization patterns, SLE patients were significantly more costly in each category. The highest cost difference was inpatient costs, where costs of SLE patients were twice of these of non-SLE patients ($13,795 versus $6,660, *P* < 0.001). The total cost were $9,238 higher for the SLE patients than for their matched controls ($29,232 versus $19,994, *P* < 0.001). 

### 3.3. Multivariate Analysis Results

Multivariate regressions were conducted to estimate the marginal impact of having SLE on healthcare utilization and costs, controlling patients' demographic and clinical characteristics between matched SLE and non-SLE patients. Logistic regressions estimated an odds ratio of 2.6 for having at least one inpatient admission and 2.0 for having at least one ER visit per year for SLE patients relative to their controls. After GLM model adjustment, SLE patients had 0.3 more inpatient admissions, 0.7 more ER visits, 3.5 more physician office visits, 1.2 more hospital outpatient visits, and 15.9 more other outpatient services per year than their matched controls (*P* < 0.001 in all cases). GLM models also estimated that SLE patients had $10,984 more total cost, $5,890 more inpatient costs, $2,418 more outpatient costs, and $1,160 more outpatient pharmacy costs per year than non-SLE patients (*P* < 0.001 in all cases, Figure 2), holding everything else constant. 

### 3.4. SLE Flares and Costs

Flares were very common with 97% of SLE patients experiencing at least one flare (75% experiencing mild, 91% moderate, and 25% severe) during the mean followup of 38.8 months (Table 3). On average, each patient had 0.9 mild flares, 1.6 moderate flares, and 0.1 severe flares per year, or 2.8 mild, 5.2 moderate, and 0.4 severe flares per patient during the entire followup period. Severe flares had the highest cost per flare episode ($11,716, SD $29,141), followed by moderate flares ($562, SD $2,275) and mild flares ($129, SD $702). Most of the cost difference between severe flare episodes and moderate or mild flare episodes was inpatient costs, as by definition there was no SLE-related hospitalization in moderate or mild flare episodes.

Annual total medical costs, whether SLE associated or not, increased with the highest severity level of flares patients experienced during the followup period: $49,754 ($SD 81,286) for patients with severe flares as the highest flare severity versus $21,941 ($SD 40,583) for patients with moderate flares as the highest flare severity versus $17,574 ($SD 40,333) for patients with mild flares as the highest flare severity. Patients with severe and moderate but no mild flares and patients with severe flares only incurred the highest annual cost ($66,412, $SD 101,704, and $74,491, $SD 64,204, resp.).

## 4. Discussion

In this retrospective study of US Medicaid enrollees, SLE patients had significantly higher healthcare utilization and higher overall expenditures than patients with no SLE. Compared with a matched cohort of patients without SLE, SLE patients incurred $10,984 more total cost per year with 55% of that being attributed to inpatient care. This was consistent with the $12,238 incremental cost estimated by Carls et al. in SLE patients with commercial insurance [7]. It is also within the range of $3,735–$14,410 for the average annual direct costs of SLE, as estimated in 11 studies covering US, Canada, Germany, UK, and Hong Kong [18]. Our incremental cost was higher than the estimate from Li et al. who found an incremental cost associated with SLE ranging between $3,795 and $8,189 in each of the first five years from the first SLE diagnosis in Medicaid patients [8]. This difference can be explained as the following. First, Li et al. required all study patients to have at least 5 years of continuous enrollment, while our study only required at least 1 year of continuous enrollment. If we only include the subset of patients with at least 5 years of followup, the incremental costs would be $7,540, consistent with the estimates from Li et al. Second, Li et al. only examined newly active SLE patients, while we included both incident and prevalent SLE patients. Last, our study conducted multivariate regressions to adjust for remaining unbalances in patient characteristics after matching, and Li et al. did not. 

Nearly all SLE patients (97%) experienced at least one flare during the 39-month followup. Although the number of severe flares per patient per year was lower than the number of moderate and mild flares, severe flares had the highest attributable cost per episode, which was 20 times higher than that of moderate flares and 90 times higher than that of mild flares. A study of commercially insured SLE patients also found cost per episode higher for severe flares than for moderate and mild flares (severe: $17,059; moderate: $1,539; mild: $909), and their cost per episode was higher than ours in all episodes [19]. Patients who experienced severe flares (25% of the sample during followup) also incurred the highest annual total costs compared to patients with less severe flares. In addition, at each flare severity level, annual total costs in this Medicaid population were higher than those in a commercially insured SLE population (patients with severe flares: $$36,214 in commercial versus $49,754 in Medicaid; moderate: $11,125 versus $21,941; mild: $5,562 versus $17,574). Because flares, especially severe flares, represent a significant economic burden in SLE patients, SLE treatment that prevents or reduces severe flares may generate significant cost savings. 

It is worth noting that many SLE patients had multiple comorbid conditions. Three in ten SLE patients had hypertension, one in four had cardiac disease, and more than one in ten had pulmonary disease, depression, anemia, and rheumatoid arthritis. Medication reconciliation may be needed in the treatment of SLE and comorbidities.

Several limitations should be considered when interpreting these study results. First, like all administrative healthcare claims databases, MarketScan databases rely on administrative claims data for clinical detail. The diagnosis of SLE and comorbid conditions was determined exclusively based on diagnosis and procedure codes. Although the definition of SLE in this study has been used in several published studies, [5, 7, 8] it cannot be confirmed due to the lack of chart review data. In addition, since comorbidities were defined during a 6-month preperiod, the proportion of patients with specific comorbid conditions could be higher if a longer preperiod was used. Second, costs for patients with capitated services (30% of the study patients) were imputed based on the mean costs of noncapitated claims by state or health plan, age grouping (17, 18–34, 35–44, 45–54, 55–64, 65–74, 75–84, and 85+), and major diagnostic category for inpatient claims, and state or health plan, age grouping, and procedure group for outpatient claims. Thus, costs of patients with capitated services may not reflect the actual costs incurred. Last, because our analysis was based on a population covered by Medicaid in 10 states, cost burden to the entire Medicaid population was not estimated, and the results from our study may not be generalizable to patients in Medicare, those with commercial insurance or who are uninsured.

Despite these limitations, this study provides cost estimates associated with SLE and SLE flares in a Medicaid population in the US based on the most recent data available. This information will help Medicaid programs understand true direct medical care costs associated with SLE.

## 5. Conclusion

SLE significantly increases healthcare utilization and costs. The incremental annual cost associated with SLE was estimated as $10,984, 55% of which was associated with inpatient costs. SLE flares were experienced by 97% of SLE patients, with an average of 2.6 flares per patient per year. Cost per flare was highest for severe flares at $11,716. Patients with at least one severe flare during the followup period had an annual cost of $49,754, more than twice the costs of patients with moderate or mild flares as their highest flare severity. Further research is needed to understand the potential impact of various SLE treatments on healthcare utilization and costs in the Medicaid population.

## Figures and Tables

**Table 1:** Demographic and clinical characteristics of SLE and non-SLE patients.

	Before matching			Postmatching		
	SLE patients	Non-SLE patients	Standardized difference^†^	SLE patients	Non-SLE patients	Standardized difference^†^
*N *	14,777	341,182		14,262	14,262	
Female	92.8%	66.9%	68.2	92.6%	92.4%	0.6
Mean age (SD)	45.4 (14.3)	48.3 (20.4)	−16.6	45.4 (14.4)	48.0 (14.9)	−17.5
Insurance plan type: fee for service	70.6%	69.7%	2	70.5%	72.3%	−3.9
Medicare dual eligibility	39.7%	39.8%	−0.2	39.6%	43.2%	−7.4
Race						
White	39.1%	41.1%	−4.2	39.4%	40.6%	−2.4
Black	36.6%	21.0%	35	36.1%	34.6%	3.2
Hispanic	11.4%	21.6%	−27.8	11.5%	10.7%	2.7
Other/missing	13.0%	16.3%	−9.4	13.0%	14.2%	−3.5
Basis of eligibility						
Aged (≥65 years)	6.5%	20.6%	−42.2	6.7%	7.6%	−3.3
Blind/disabled	64.6%	36.0%	59.5	63.9%	67.6%	−7.9
Adult	22.0%	36.0%	−31.3	22.5%	19.0%	8.7
Other	6.9%	7.3%	−1.4	6.9%	5.8.0%	4.5
Length of followup (months, mean, SD)	38.8 (20.4)	33.6 (18.1)	26.57	38.7 (20.3)	38.0 (20.2)	3.72
Charlson-Deyo comorbidity index (mean, SD)	1.16 (1.39)	0.41 (1.01)	61.9	1.11 (1.33)	1.26 (1.80)	−9.4
Comorbid conditions						
Rheumatoid arthritis and other inflammatory polyarthropathies	10.6%	0.7%	43.6	8.7%	7.6%	4.1
Autoimmune thyroid disorders	0.1%	0.0%	3.7	0.1%	0.1%	0.9
Anemia	14.2%	3.4%	39.1	13.2%	13.4%	−0.6
Pericarditis	0.3%	0.0%	6.5	0.2%	0.1%	1.2
Raynaud's syndrome	1.2%	0.0%	14.9	0.7%	0.4%	3.2
Thrombocytopenia	1.8%	0.2%	16.6	1.6%	1.4%	1.7
Myositis	9.8%	1.3%	37.9	8.9%	8.5%	1.4
Hypertension	30.2%	15.9%	34.5	29.2%	32.1%	−6.5
Renal disease	7.5%	1.8%	27.3	6.9%	6.7%	0.7
Depression	15.7%	8.0%	23.8	15.3%	16.1%	−2.1
Cardiac disease	24.3%	10.8%	36.2	23.3%	25.3%	−4.6
Cerebrovascular disease	4.8%	2.6%	11.5	4.7%	4.9%	−1.2
Liver disease	2.1%	0.8%	11	2.0%	2.2%	−1.2
Pulmonary disease	17.5%	8.3%	27.6	17.0%	18.9%	−4.8
Nephritis*	4.7%	0.5%	26.7	4.1%	3.5%	3.4
Concomitant medications of interest**	3.5%	2.2%	7.7	3.5%	3.3%	0.8

^†^The absolute value of standardized difference <10 is a good match.

*Nephritis was not included in renal disease.

**Concomitant medications include hydralazine, quinidine, procainamide, phenytoin, isoniazid, and d-penicillamine.

**Table 2:** Annualized healthcare utilization in the followup period for SLE patients and their matched controls.

	SLE patients		Non-SLE patients		Difference	*P* value
	*N* = 14,262		*N* = 14,262			
Number of patients with at least 1 inpatient admission during the entire followup (*N*, %)	8,459	59.3%	5,644	39.6%	20%	<0.001
Among those with at least 1 IP admission, number of inpatient admissions (mean, SD)	1.2	1.6	1.0	1.3	0.2	<0.001
Number of inpatient admissions among all patients (mean, SD)	0.7	1.3	0.4	1.0	0.3	<0.001
Number of patients with at least 1 ER visit during the entire followup (*N*, %)	11,202	78.5%	9,494	67.5%	11%	<0.001
Among those with at least 1 ER visit, number of ER visits (mean, SD)	2.6	4.7	2.1	3.5	0.6	<0.001
Number of ER visits among all patients (mean, SD)	2.1	4.3	1.4	2.9	0.7	<0.001
Number of physician office visits (mean, SD)	9.8	8.2	6.4	7.0	3.4	<0.001
Number of outpatient hospital visits (mean, SD)	3.3	5.8	2.2	5.0	1.1	<0.001
Number of other outpatient services (mean, SD)	69.7	86.8	56.8	89.8	12.9	<0.001

*N*: number, IP: inpatient admission, ER: emergency room.

**Table 3:** Number of flares and cost per flare.

	Mild flare		Moderate flare		Severe flare	
Number of flares per patient per year (mean, SD)	0.9	1.0	1.6	1.2	0.1	0.3
Number of patients with at least 1 flare during the entire followup (*N*, %)	10,696	75%	13,002	91%	3,540	25%
Number of months of followup (mean, SD)	40.5	20.5	39.8	20.5	43.0	21.9
Number of flares per patient (mean, SD)	2.8	3.0	5.2	4.5	0.4	0.9
Number of patients with only 1 flare (*N*, %)	2,663	25%	1,608	12%	2,666	75%
Number of patients with only 2 flares (*N*, %)	2,110	20%	1,698	13%	464	13%
Number of patients with 3+ flares (*N*, %)	5,923	55%	9,696	75%	410	12%

Cost per flare	Mean (SD)	Median	Mean (SD)	Median	Mean (SD)	Median

Medical cost	$45 ($416)	$0	$477 ($2,221)	$69	$11,621 ($29,148)	$2,138
Pharmacy cost	$84 ($562)	$25	$84 ($506)	$5	$94 ($488)	$8
Total cost	$129 ($702)	$36	$562 (2,275)	$123	$11,716 ($29,141)	$2,294
